# More may not be better: reconsidering early clinical trial design in the era of targeted cancer therapeutics

**DOI:** 10.1002/1878-0261.13391

**Published:** 2023-02-15

**Authors:** René Bernards

**Affiliations:** ^1^ Division of Molecular Carcinogenesis, Oncode Institute The Netherlands Cancer Institute Amsterdam The Netherlands

**Keywords:** drug development, phase 1 trials, targeted cancer drugs

## Abstract

The advent of a new generation of targeted cancer drugs requires a radically different design of early clinical trials. Here, René Bernards discusses why new clinical trial designs are needed and what is being done to achieve this. Such innovative trials can lead to similar outcomes for cancer patients with fewer side effects while at the same time reducing the cost of cancer care.

## Graphical abstract

In a series of Viewpoints, EACR Board members present their thoughts on current topics in cancer research. Here, René Bernards discusses why new clinical trial designs are needed and what is being done to achieve this. Such innovative trials can lead to similar outcomes for cancer patients with fewer side effects while at the same time reducing the cost of cancer care.

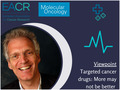

**Abbreviations**
FDA: Food and Drug Administration
MDICT: methodology for the development of innovative cancer therapies
MTD: maximum tolerated dose
RDR: recommended dose range
RP2D: recommended phase 2 dose

Over the past decades, advances in our understanding of the molecular basis of cancer have resulted in drugs that are fundamentally different from the conventional chemotherapies that predate this revolution. Targeted cancer therapeutics selectively inhibit the oncoproteins that drive cancer proliferation and survival or stimulate the immune system to kill cancer cells [1]. Despite this paradigm shift in the treatment of cancer, the early clinical development of oncology drugs has barely changed. When cytotoxic chemotherapies were first developed, it was generally assumed that drug toxicity is closely linked to drug efficacy. This resulted in a clinical trial design in which drugs were initially given in increasing doses until toxicity became limiting (phase 1 trials). Drug concentrations close to this ‘Maximum Tolerated Dose’ (MTD) became the Recommended Phase 2 Dose (RP2D) used in subsequent phase 2 clinical studies designed to see signs of efficacy. It is becoming increasingly evident, however, that for targeted cancer drugs, the adage ‘MTD ≈ RP2D’ no longer applies. Targeted cancer therapeutics tend to display significant selectivity for a mutant protein over the nonmutated counterpart found in normal cells, creating a larger ‘therapeutic window’—a drug concentration range in which the drug selectively affects cancer cells. Moreover, for mutant‐selective drugs, the toxicity seen in the clinic may not be ‘on‐target’, making toxicity a rather unsophisticated endpoint for phase 1 studies. As a case in point, for some of the most recent targeted cancer drugs, the MTD was not reached in phase 1 [2]. It should be noted that this holds less true for the relatively unselective multikinase inhibitor drugs, where off‐target effects can be significant.

So, what should be done instead? Various publications have highlighted the need for change in phase 1 trial design [3, 4], and several initiatives have been taken to address this issue. In Europe, the task force on Methodology for the Development of Innovative Cancer Therapies (MDICT) was established in 2006 to consider new paradigms for phase 1 studies with targeted therapies [5], which recently published its guidelines [6]. In the United States, the Food and Drug Administration (FDA) has launched project Optimus, which aims to optimize the dose selection for oncology drugs [7]. The recommendations from these efforts cannot be summarized in a single sentence, but the need for markers of target engagement (pharmacodynamic biomarkers) is highlighted and the need to define a recommended dosage range (RDR) for phase 2, rather than a single RP2D. While these developments are encouraging, it is at the same time sobering to realize that imatinib (being the first targeted cancer drug) was already approved by the FDA in 2001.

Another case of ‘more may not be better*’* concerns the length of drug treatment after cancer surgery (adjuvant therapy). Drugs like trastuzumab (which targets HER2) are typically given for 12 months postsurgery for HER2‐positive breast cancer, but a recent trial showed that 6‐month treatment was noninferior to 12 months but was associated with less cardiotoxicity and fewer severe adverse events [8]. Similarly, checkpoint immunotherapies are often given in the adjuvant setting for a year or more, while recent trials in which these drugs are given prior to surgery (neoadjuvant) indicate major and durable responses after only 6 weeks of treatment for patients with melanoma [9]. This begs the question whether lengthier (and more costly) treatment postsurgery is justified.

Studies that aim to reduce drug treatment length or dosing are difficult to finance, given the lack of financial incentive for the pharmaceutical industry to perform such studies. Clearly, the benefit of such noninferior treatments lies with the patients that may suffer fewer side effects and with the health insurance companies that can save costs and get better overall health outcomes. How should future studies like the above‐mentioned 6‐ *versus* 12‐month trastuzumab be financed? I argue for the establishment of a revolving fund, with initial start‐up funding from health insurance companies. When a study shows the noninferior benefit from shorter treatment (or using a lower drug dose), part of the savings will go to the health insurance companies and another part is re‐invested in new studies with a similar aim. Over time, this will lead to better overall health outcomes at lower cost to society. Who can be against that?

## Conflict of interest

The authors declare no conflict of interest.
